# Design and testing of an MRI-compatible cycle ergometer for non-invasive cardiac assessments during exercise

**DOI:** 10.1186/1475-925X-11-13

**Published:** 2012-03-18

**Authors:** Silmara Gusso, Carlo Salvador, Paul Hofman, Wayne Cutfield, James C Baldi, Andrew Taberner, Poul Nielsen

**Affiliations:** 1 Liggins Institute, University of Auckland, Auckland, New Zealand; 2 Department of Engineering Science, University of Auckland, Auckland, New Zealand; 3 Department of Medicine, University of Otago, Otago, New Zealand; 4 Auckland Bioengineering Institute, University of Auckland, Auckland, New Zealand; 5 Liggins Institute, University of Auckland, 2-6 Park Ave, Grafton, Auckland, New Zealand

**Keywords:** Cycle ergometer, Left ventricular function, Magnetic resonance imaging

## Abstract

**Background:**

Magnetic resonance imaging (MRI) is an important tool for cardiac research, and it is frequently used for resting cardiac assessments. However, research into non-pharmacological stress cardiac evaluation is limited.

**Methods:**

We aimed to design a portable and relatively inexpensive MRI cycle ergometer capable of continuously measuring pedalling workload while patients exercise to maintain target heart rates.

**Results:**

We constructed and tested an MRI-compatible cycle ergometer for a 1.5 T MRI scanner. Resting and sub-maximal exercise images (at 110 beats per minute) were successfully obtained in 8 healthy adults.

**Conclusions:**

The MRI-compatible cycle ergometer constructed by our research group enabled cardiac assessments at fixed heart rates, while continuously recording power output by directly measuring pedal force and crank rotation.

## Background

Magnetic Resonance Imaging (MRI) and echocardiography are the most common non-invasive methods for assessing left ventricular function and structure. Previous studies have highlighted the advantages of MRI over echocardiography [1,2]. MRI scanning allows for three-dimensional estimation that is not affected by preload conditions, geometric assumptions, or the skill of the operator [3]. The use of MRI technology provides clear definitions of endocardial and epicardial borders, allowing an accurate and reproducible evaluation of left ventricular mass and volume throughout the cardiac cycle. For these reasons MRI scanning is considered the "gold standard" for cardiac function and structure evaluation.

Magnetic resonance imaging has become an important tool for cardiac research, and resting cardiac assessments are now routinely performed. Dobutamine stress tests during MRI scanning are commonly used to evaluate the cardiac function at a target heart rate [4]. However, this is an invasive procedure with risk of severe side effects and therefore not always suitable for a research environment, especially if the research involves children and adolescents [4]. Research into MRI-based exercise cardiac evaluation is limited [5-9], even though it can unmask abnormalities that are not seen at rest. Commercially available ergometers use 'fixed workloads', where the pedalling resistance is maintained regardless of pedalling frequency. This method provides the most accurate assessment of myocardial responses to a given external stress, but is not effective in controlling the heart rate at which images can be obtained [10]. In addition, exercising within the narrow diameter MRI bore is an issue. Leg movement can be restricted, making it difficult to position the chest far enough into the MRI scanner, consequently hindering image quality. Although several MRI-compatible exercise instruments have been built and tested, none provide continuous workload measurement, allowing accurate regulation of exercise heart rate [5,9,11-13]. Few studies have aimed to assess the cardiac response to exercise using MRI technology, and the majority have used commercially available MRI-compatible cycle ergometers at fixed workloads to evaluate left ventricular function and structure [5,7-9,14]. Studies on diastolic function would also benefit from stable heart rates and controlled cardiac cycle duration. Thus, cardiac MRI assessments during exercise at fixed heart rate would be a useful modality.

Thus, we aimed to design a portable and affordable MRI cycle ergometer that continuously measures power, through the direct measurement of force and pedal rotation while patients exercise to maintain target heart rates. The continuously adjustable workload measurement should be capable of maintaining target heart rate, with minimum variability. For this purpose, we aimed to design an ergometer that would: a) provide variable resistance to control workload; b) allow patients to be positioned close to the MRI isocenter. We report here a custom-built MRI-compatible cycle ergometer that meets the above requirements, as well as an associated exercise protocol, both designed by our research group for left ventricular evaluation.

## Methods

### MRI cycle ergometer

The MRI cycle ergometer was designed to function without interfering with the scanned images. Apart from the above described requirements, it was necessary for the ergometer to: 1) accurately measure power output (up to 200 W); 2) be compact; 3) have minimal initial static and kinetic friction; 4) be easy to set up and install; 5) function over the normal range of men and women's heights; 6) have a comfortable and secure feet placements; 7) ensure minimal electronic interference on MRI image quality; 8) continuously record forces and displacements; and 9) offer resistance control.

#### Ergometer

**Figure 1:**
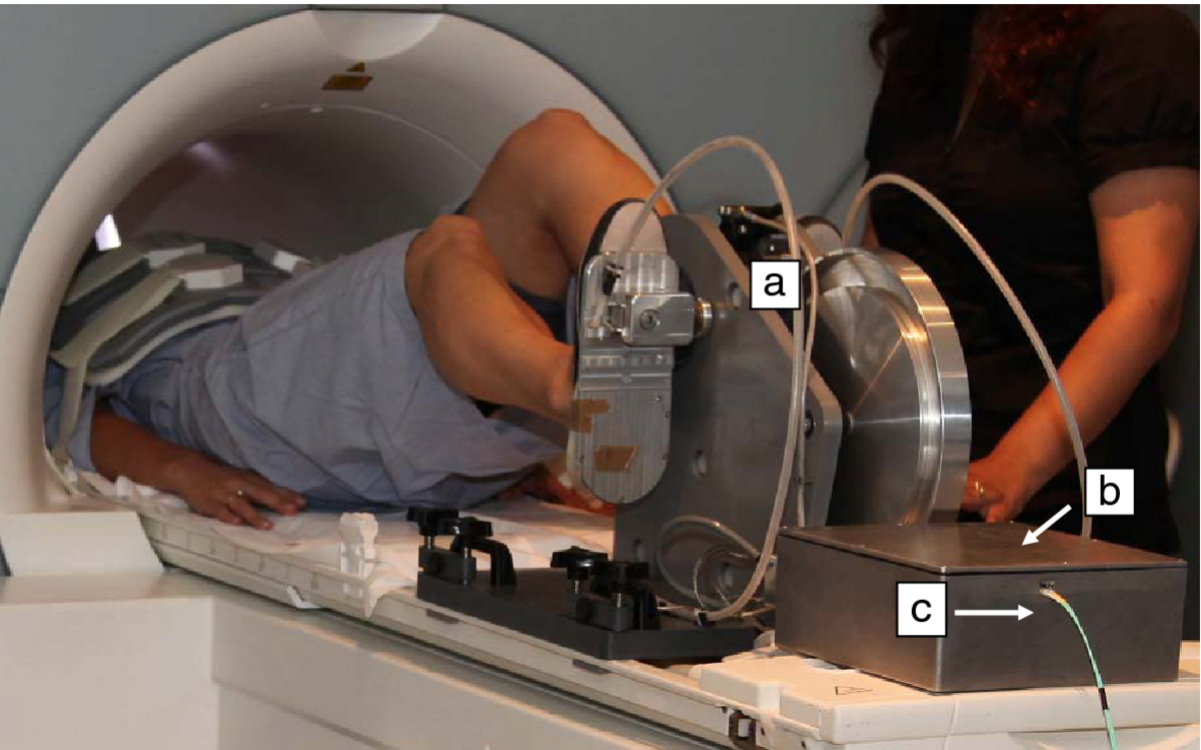
**MRI cycle ergometer setting**. Volunteer prep at MRI room with cycle ergometer (**a**) electronic box, (**b**) and optical fibre line, (**c**) before the MRI bed is moved to isocentre.

**Figure 2:**
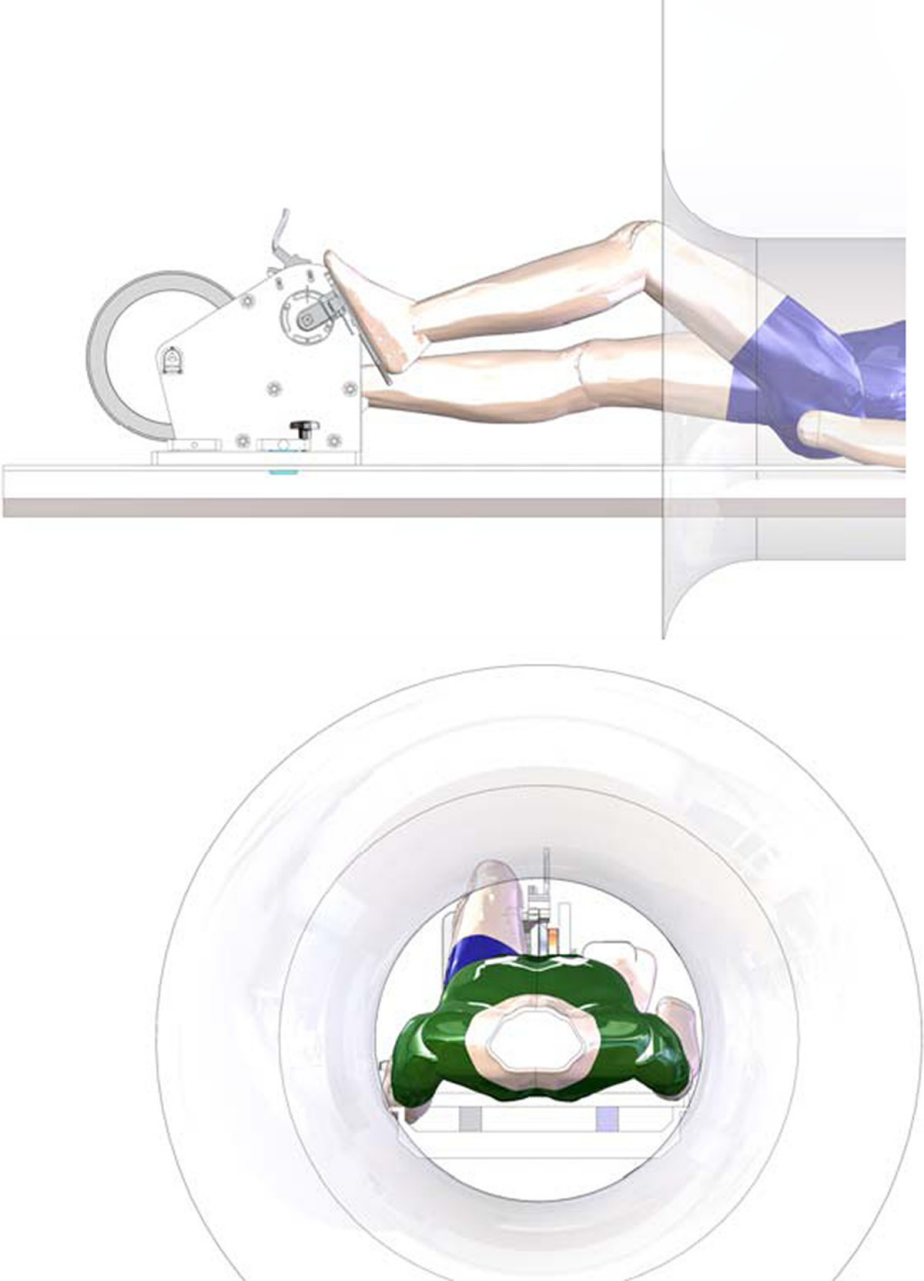
**Layout of ergometer, MRI, and patient**. Relative placement of the ergometer, MRI and patient. The position of the ergometer can be adjusted to accommodate patients of various heights and leg-torso lengths.

The cycle ergometer was specifically made to fit into a 1.5 T MRI scanner with a 600 mm bore (Magneto Avanto; Siemens, Erlangen, Germany), and consists of an aluminium pedal system with two force transducers, a pulley and an aluminium flywheel, a hydraulic disk brake, a rotary position encoder, and an electronic enclosure (Figure 1). The ergometer was designed to be positioned at the end of the MRI bed, firmly screwed on the bedside with a polyvinylchloride trunnion and base plate (Figure 1). A crank length of 60 mm (Figure 2) was used to allow for subjects of different heights (typically 1.55 m to 1.85 m), torso, and leg length to be positioned comfortably, with the heart within 110 mm of the MRI isocenter in order to obtained optimal images. This was established in accordance to manufacturer's application specialist recommendations that optimal position should be as close to the isocenter as possible, and that beyond 150 mm off resonance artefact are obtained. The foot pedal was designed to accommodate a range of foot sizes, and included foot-straps to secure the feet in retraction. The complete ergometer weighed 27 kg, and it was 390 mm long, 550 mm wide, and 385 mm high. Mean patient power output was modulated by the MRI operator using a manually adjustable break control located in the MRI control room. Note that care was taken to avoid the use of ferromagnetic materials. A 3D PDF model of the ergometer is provided as supplementary material.

#### Position measurement

A three channel optical encoder (HEDS-5540, Avago Technologies) was used to measure the angular position (A_c_) and angular velocity (ω) of the crank. The position encoder returned a two-channel quadrature output to indicate angular motion and direction, and an additional once-per-revolution index pulse. Encoder signals interfaced with a digital position counter on a data acquisition device. The position encoder was enclosed by a customised aluminium casing to reduce electromagnetic and radio-frequency interference (EMI/RFI).

#### Force measurement

In order to calculate power, the force applied by the subject to each foot pedal was measured. An MRI-compatible force transducer (Futek model LRF350, arranged in full-bridge configuration) was integrated into each pedal to measure the extension and retraction forces (F_f_) applied by each foot. Bridge signals were fed-through to the electronic enclosure, where an instrumentation amplifier (INA128, Texas Instruments, gain = 518) amplified and filtered the signals prior to digitisation. A Sallen-Key hardware filter in low pass configuration (cut-frequency of ~100 Hz, gain of 1.53) was also implemented in the circuit. The force transducers were calibrated using a mechanical testing system (Instron 5800 series) with a 10 kN load cell. Three calibration trials were conducted on each force transducer. The force transducers exhibited a high degree of linearity (R^2 ^= 0.999) over the specified force measurement range, with sensitivity of 14 μV/N.

#### Data acquisition

All electrical signals were transmitted through shielded cables to an electronic enclosure (Figure 1b), which consisted of a die-cast aluminium-alloy box (250 mm long, 250 mm wide, 100 mm high, and 3 mm wall thickness) that provided EMI/RFI shielding properties. The enclosure contained a rechargeable lead-acid battery (10 Ah, 6 V) printed circuit boards, data acquisition device and USB fibre-optic interface. As the electrical signals entered the electronic enclosure, they were passed through signal conditioning circuitry that attenuated radio frequency interference and low-gradient magnetic pulse noise. Analog signals were then digitised and collected using a USB-6122 (National Instruments, Austin, USA) data acquisition device. A fibre-optic interface was used to convert the digital Universal Serial Bus signals to optical signals, which were transmitted via optical fibre (Figure 1c) to an interface and computer located in the MRI operator room.

#### Software design

The pedal crank was rotated to the horizontal position and the patient's feet securely attached to the pedals. An initialisation software routine was triggered, zeroing out the weight of the foot acting on each transducer, and creating a reference-point position of the crank for use in subsequent analysis.

**Figure 3:**
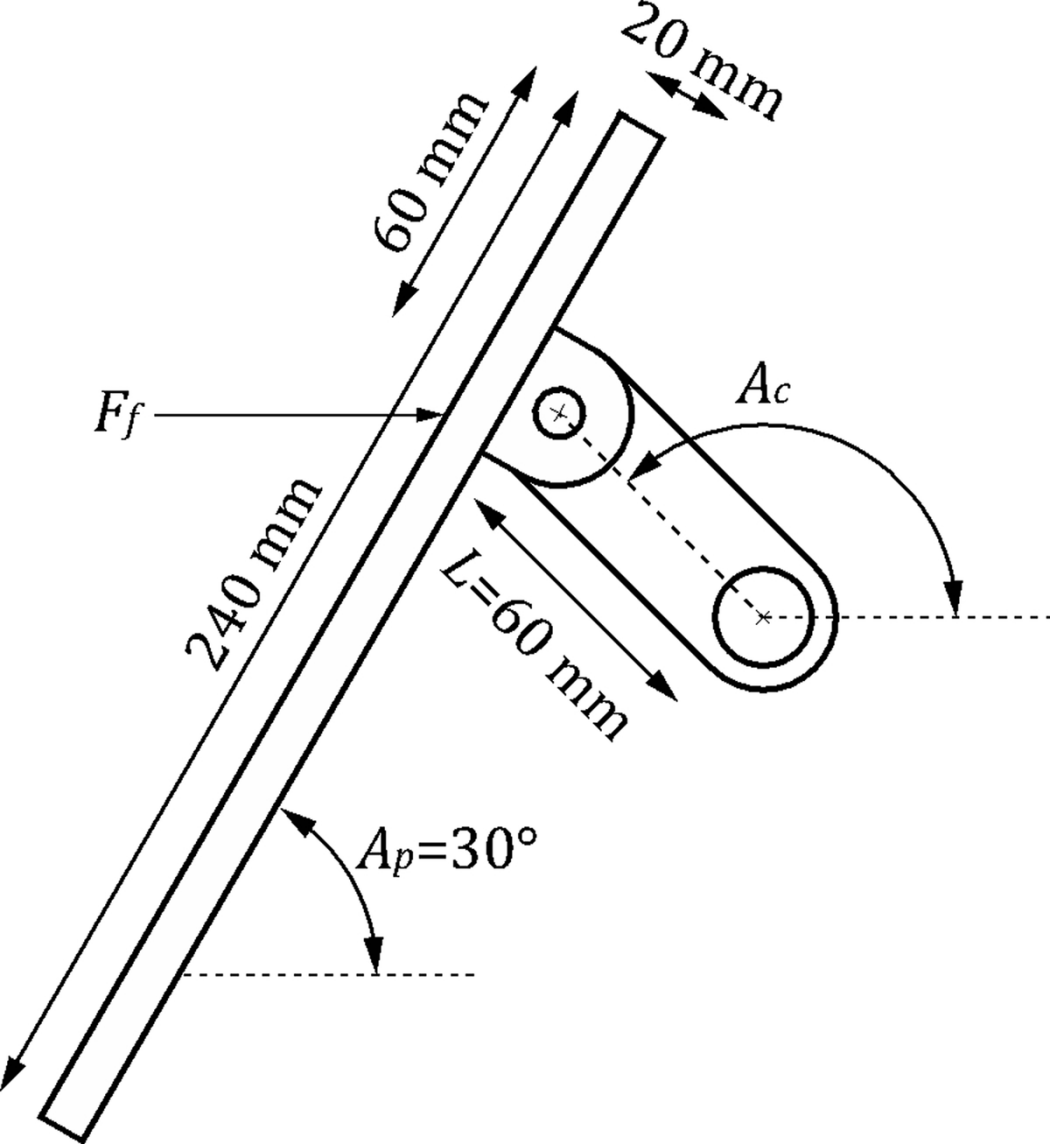
**Single ergometer pedal and crank**.

Estimates of patient instantaneous power output were computed from the acquired force and position signals as follows. Each force transducer measures a force *F_t _*= *F_f _*sin*A_p_*, where *F_f _*is the horizontal force applied by the foot to the pedal, and *A_p _*is the angle between the footplate and the horizontal (Figure 3). The component of *F_f _*that is normal to the crank creates a torque *τ *on the crank given by t = -*F_f _LsinA_c_*, where *L *is the length of the crank. This torque is related to the measured force (*F_t_*) by $t=-F_{t}L\frac{\text{sin}A_{c}}{\text{sin}A_{p}}$.

When a patient is lying in the MRI machine with their feet attached to the footplates, we observe that *A_p _*is approximately 30° throughout each rotation of the crank; we therefore assume this value for subsequent calculations. By summing the torques from each crank (yielding the net torque *τ_n_*) we compute the power (*P*) imparted by the patient to the ergometer as = *t_n_ω*.

**Figure 4:**
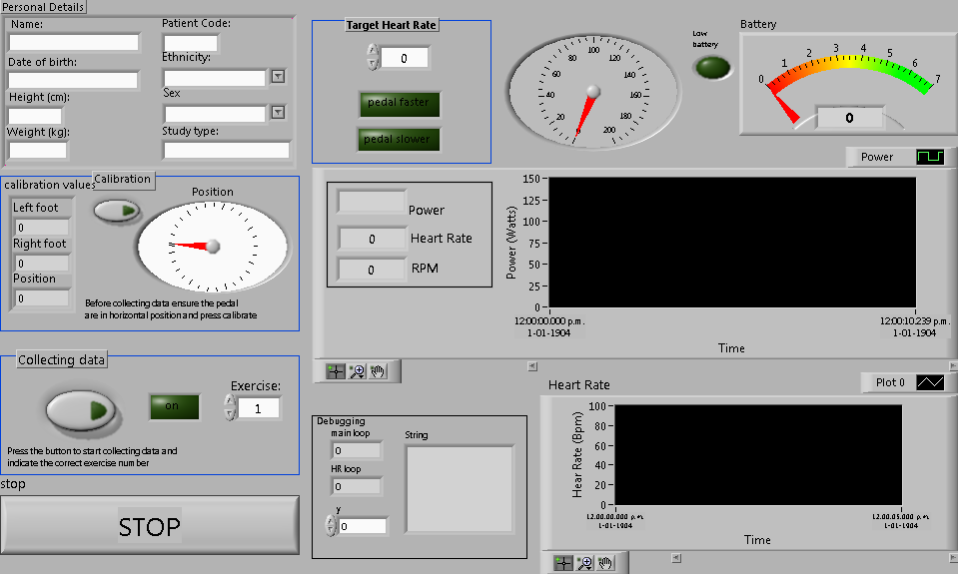
**Ergometer software interface**.

A software interface (Figure 4) for the ergometer was designed in the LabVIEW 8.5 programming environment. Software was created to provide the MRI operator with regular updates of crank angle, angular velocity, force, battery voltage, and patient power generation. Power estimates were low-pass filtered, before being displayed on-screen, in order to provide a smooth update to the MRI operator. Patient heart rate was sensed using the MRI machine's heart rate monitor (Invivo Magnitude 3150, Florida, USA), and transmitted wirelessly in RS232 format to the computer in the MRI control room. The software interface allowed patient details to be entered and recorded in a log file together with all data acquired throughout the experiment. Pedal force and crank position signals were acquired and recorded to disk at a rate of 1 kHz. Heart rate was recorded at a rate of 1 Hz.

### Participants' recruitment and baseline data

Eight healthy sedentary volunteers (4 males and 4 females, aged 20 year to 30 year) were recruited to test the MRI ergometer and exercise protocol. This protocol was approved by the Northern X Regional Ethics Committee, Auckland, New Zealand (NTX/07/12/125). All participants provided written consent and completed a health screen questionnaire prior to assessments. Body composition and fitness level were assessed one week prior to the cardiac MRI scanning. Total body scans were performed by Dual-Energy X-ray Absorptiometry (DEXA; GE Lunar Prodigy, Madison, USA). Standard manufacturers' software was used to determine fat mass, fat free mass and body fat percentage. Anthropometric data obtained from all participants included weight, height and body mass index (BMI).

Maximal oxygen consumption (VO_2max _test) was measured by the patient pedalling to exhaustion on an electronically-braked cycle ergometer (Schiller, Switzerland). The exercise protocol consisted of a 3 minute to 5 minute warm-up at a low workload (20 W to 40 W) followed by successive one-minute stages starting at 55 W, incrementing 15 W per stage. Breath-by-breath data were collected and analyzed using a ParvoMedics TrueOne 2400 Metabolic Measurement System (Parvomedics, Sandy, USA) calibrated with room air and standardized gas. The rate of oxygen consumption (VO_2_) and carbon dioxide production (VCO_2_) were recorded every 30 s. The average of the two highest consecutive VO_2 _values was defined as VO_2max_. The test was considered a true maximum if either a plateau or an increase of less than 250 ml in VO_2 _occurred in spite of an increase in workload or a respiratory exchange ratio (RER) greater than 1.1 was achieved. Blood pressure was recorded both at the beginning and termination of the test.

### Cardiac MRI protocol

Resting and exercise cardiac scans were performed on a 1.5 T Magnetom Avanto (Siemens, Erlangen, Germany) scanner using a 12 channel body matrix coil in combination with the spine matrix coil. Images were acquired with retrospective ECG-gating (Invivo Magnitude 3150 System, Orlando, USA). The MRI-compatible cycle ergometer was positioned at the end of the scan table, and fibre optic cable fed through the waveguide to outside computer. Once a participant was positioned on the MRI table, electrodes were attached to the anterior chest wall and a blood pressure cuff was placed on the left arm (Invivo Magnitude, Florida, USA). Headphones and an emergency buzzer (right hand) were also provided. The participant's feet were then strapped into pedals and their body position adjusted on table. A velcro strap was also positioned across the participant's hip to prevent upwards movement on the bed due to leg motion during cycling, In addition, a strap, attached to the left base of the ergometer, was given to each participant to hold (left hand) to ensure trunk stability during exercise. The participant then performed a short bout of unloaded exercise to gain familiarity with supine cycling and the breath hold manoeuvre. Once the ergometer initialisation was performed, the MRI table was advanced so that the subject's heart was located within 110 mm of isocenter, while ensuring that the participant's knees did not contact the bore during pedalling. The 1.5 T scanner used has a bore size of 600 mm. A bolster was then placed under the knees for comfort while resting images were obtained. If patient position was not at zero, the table position was reset before scanning.

#### Left ventricular function at rest

Cardiac MRI images were obtained with iPAT (integrated parallel imaging technique). Six short-axis Trufisp cine 6 mm evenly spaced images from base to apex were obtained. The distance calculation between slices was given by: (*L_v _*- 36 mm)/5 + 6 mm, where *L_v _*is the left ventricle length. Three long-axis Trufisp images were then obtained at cine 0°, 60° and 120°. Images were acquired during breath hold manoeuvres (10 s to 15 s) with phase resolution at 100% (256 × 256). Temporal resolution was set to be 30 ms. Segments were 11 - 13 and the calculate number of phases was set to a minimum of 25. Participants performed breath-hold at end-expiration during each image acquisition, in order to eliminate respiratory motion artefacts. Blood pressure was measured after all 9 images were obtained, at the end of the resting protocol.

#### Left ventricular function at sub-maximal exercise

**Figure 5:**
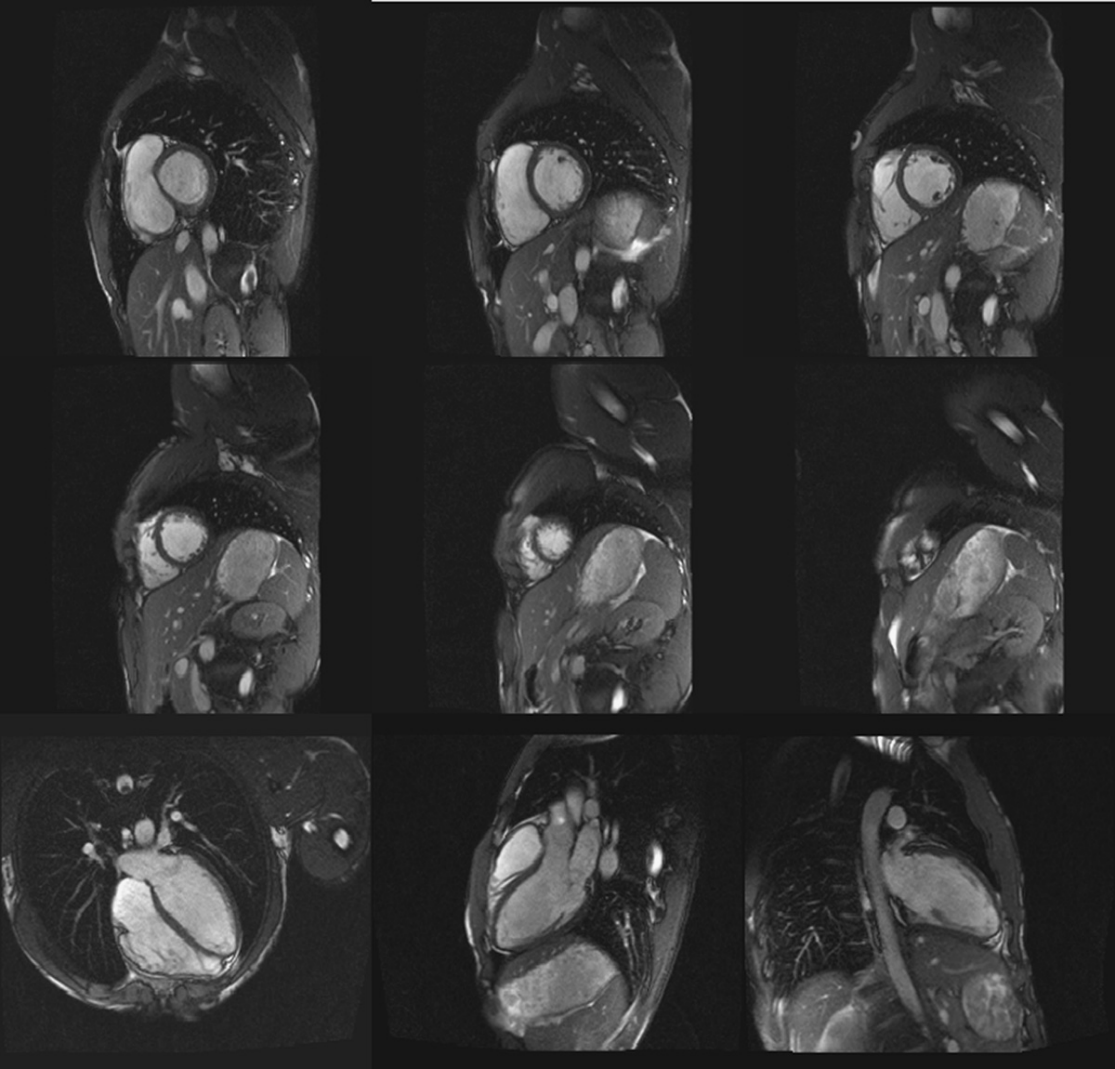
**Left ventricular images at rest**. Participant was positioned 50 mm from MRI isocenter. This image illustrates the clear definition between endocardial and pericardial borders of the left ventricle at rest on 6 short axis and 3 long axis MRI images.

**Figure 6:**
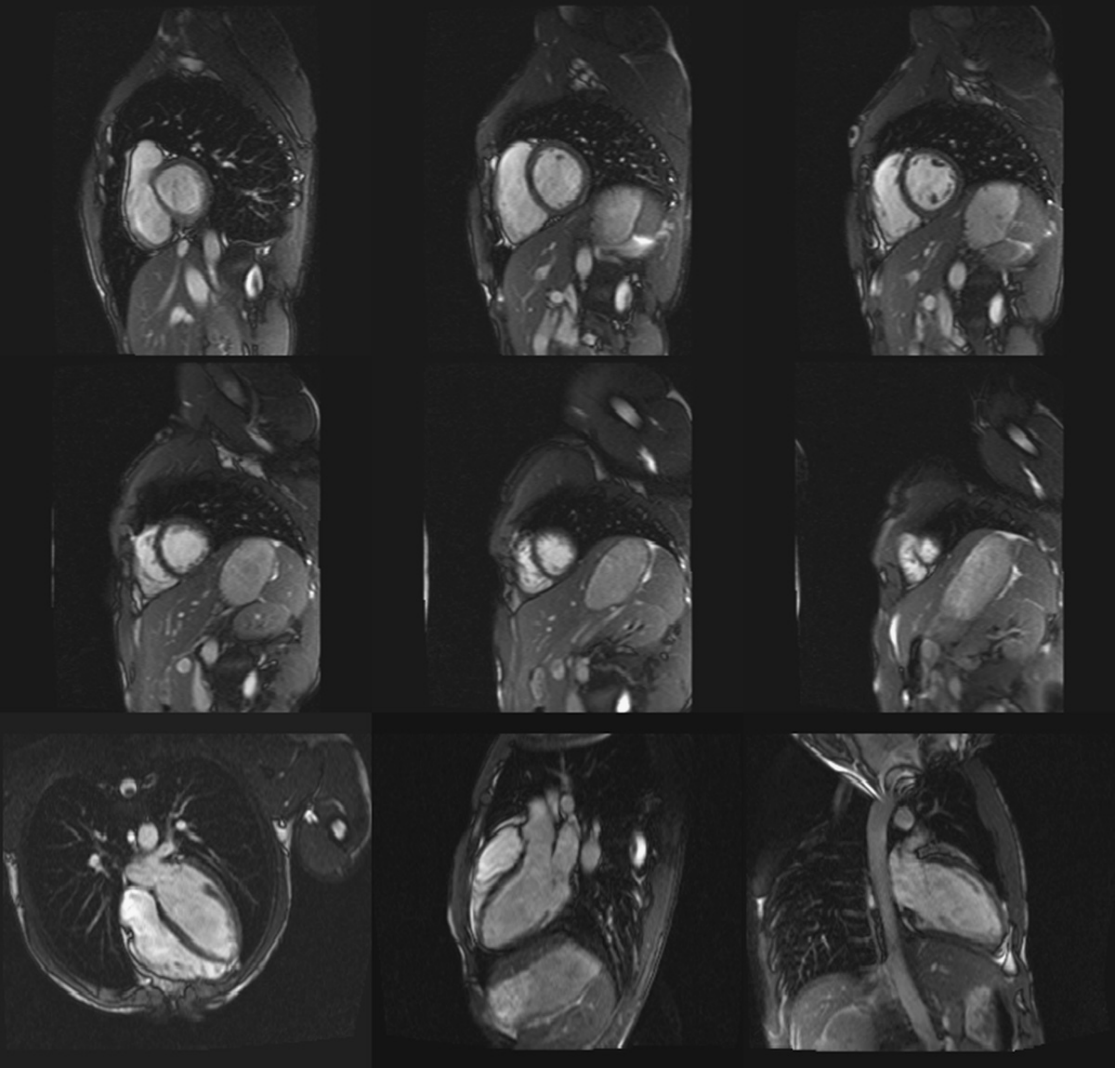
**Left ventricular images during exercise**. Participant was positioned 50 mm from MRI isocenter. This image illustrates the clear definition between endocardial and pericardial borders of the left ventricle at exercising heart rate on 6 short axis and 3 long axis MRI images.

After the resting measurements were completed, the bolster was removed and participants instructed to start pedalling. Target heart rate for this pilot study was 110 beats/min. Ergometer resistance and participant's cycling speed were adjusted accordingly to allow the target heart rate to be obtained and sustained. Left ventricular exercise images were obtained once one minute of steady state heart rate was reached. Breathing instructions were given whilst participants were pedaling, so that they would stop breathing and stop pedaling simultaneously, allowing image acquisition (5 s to 7 s). Cycling was resumed immediately afterwards. Similarly to those obtained at rest, six short-axes and three long-axis images were acquired. It was established that phase resolution could be reduced to 50% to shorten breath hold (aimed for 128 × 256), which did not degrade the quality of the images obtained (Figures 5 and 6). Sequences had 6-10 segments (max) and calculate number of phases between 20-25. Verbal feedback was constantly given to participants during exercise phase.

Cardiac MRI images analysis was performed with the CIM software (Cardiac Image Modeller, Auckland, New Zealand) using the six short-axis and three long-axis LV images. Cardiac parameters obtained included left ventricular mass, end-diastolic volume, end-systolic volume, stroke volume, cardiac output, ejection fraction (both at rest and during sub-maximal exercise). Endocardial contours of each slice were manually identified at end-diastole and end-systole for each time point through the cardiac cycle. Cardiac output was determined by multiplying stroke volume by heart rate.

### Statistical Analysis

Data obtained at rest and during exercise were compared using t-tests. The statistical package SPSS version 15.1 (LEAD Technologies Inc, USA) was used. Statistical significance was set as p < 0.05. Data are presented as mean ± standard deviation (SD).

## Results

Table 1 displays the baseline characteristics on the eight volunteers. During MRI scanning all participants were positioned within 110 mm from the MRI isocenter: two at 0 mm, two within a 50 mm distance, two within 80 mm, one at 100 mm and one at 110 mm. The variations in isocenter positioning were due to differences in participant's height and torso to leg length ratio.

**Table 1:** Baseline characteristics of participants

	Mean ± SD	Range
**N = (Female/Male)**	8 (4/4)	

**Age (years)**	25.3 ± 4	21-30

**Weight (kg)**	67.1 ± 10.2	49.8 - 83.7

**Height (m)**	1.74 ± 0.07	1.64-1.82

**BMI**	22.3 ± 3.5	18.5-30

**% body fat**	24 ± 14	6-42.8

**Fat free mass (kg)**	51.6 ± 10.4	32.5-67.3

**Resting heart rate (bpm)**	67 ± 7	57-77

**SBP - sitting (mmHg)**	104 ± 8	94-117

**DBP - sitting (mmHg)**	65 ± 4	58-70

**Max Heart Rate (bpm)**	184 ± 8	173-197

**VO_2max _(mlO_2_/kgFFM/min)**	42.9 ± 4.4	37.9-50.0

**Respiratory exchange ratio**	1.34 ± 0.09	1.1-1.4

Results are presented as mean ± standard deviation and range. SBP: systolic blood pressure; DBP: diastolic blood pressure

Cardiac results at rest and during exercise are shown on Table 2. Participant's ejection fraction, cardiac output and systolic blood pressure increased from rest to exercise, but end-diastolic volume, stroke volume and diastolic blood pressure did not. In contrast, end-systolic volume decreased in response to exercise. The quality of MRI images obtained at rest and during exercise was not different (Figures 5 and 6). Images were unaffected by magnet installation and operation, and suffer no discernible signal interference from the electronic enclosure or transducers.

**Table 2:** Cardiac function at rest and during sub-maximal exercise

	REST	EXERCISE
**Heart rate (bpm)**	67 ± 15	110 ± 3 *

**Left ventricular mass (g)**	128 ± 28	128 ± 28

**Ejection fraction (%)**	65.7 ± 3.8	74.3 ± 4.7 *

**End diastolic volume (ml)**	147.6 ± 30.6	142.4 ± 25.1

**End systolic volume (ml)**	50.9 ± 13.4	36.2 ± 10.2 *

**Stroke volume (ml)**	96.7 ± 19.1	105.7 ± 18.7

**Cardiac Output (l/min)**	6.5 ± 1.9	11.6 ± 2.1 *

**SBP (mmHg)**	108 ± 14	128 ± 22 *

**DBP (mmHg)**	66 ± 11	62 ± 6

**Workload (W)**	-	96 ± 27

Data are mean ± SD. SBP: systolic blood pressure; DBP: diastolic blood pressure. *P < 0.05 for rest vs. exercise.

## Discussion

We have successfully designed, constructed, and tested an MRI-compatible cycle ergometer for cardiac assessments, which takes into account between-subject variations, to accurately quantify workload power. The ergometer was designed to fit into a 1.5 T MRI scanner (Magneto Avanto; Siemens), and is adjustable for a relatively wide range of heights.

This ergometer has the potential to assist physiological studies looking at cardiac responses to dynamic exercise in a variety of populations. Moreover, the dose of exercise (workload) generated can be more precisely adjusted, which allows for the assessment of patients with varying degrees of fitness [10]. Our ergometer is also useful in studies targeting fixed heart rates during MRI assessments, particularly valuable when assessing cardiac function. For instance studies have shown that to predict the development of diabetic nephropathy exercise tests with fixed heart rate were preferable than fixed workload in type 1 diabetic individuals due to the differences in physiological responses such as blood pressure which were associated with the development of micro- and macroalbuminuria [15].

A number of cardiac impairments are only evident when the heart is exposed to stress. Heart rate or rate pressure product (heart rate × systolic blood pressure) have the strongest association with myocardial work of all non-invasive measures [16]. Thus, steady-state quantification of heart rate is important during cardiac function studies. An advantage of the fixed resistance bike used in this study is that, when presented with a target heart rate, the patient can increase or decrease pedalling cadence or frequency to adjust their heart rate. In contrast, the investigators must change workload or resistance for the patient when using a fixed workload ergometer.

Heart rate control is also critical in the assessment of diastolic filling properties. Peak early mitral valve inflow velocity, an indicator of diastolic function, is inversely proportional to heart rate [17]. This is largely due to the preferential reduction in diastolic duration during tachycardia [18]. Therefore, accurate comparisons of diastolic function in different groups require equal heart rates (i.e. diastolic durations). Previous investigations using fixed workload ergometers have described standard deviations of up to 17 beats per minute around the targeted heart rates [10]. In contrast, the standard deviation around our target heart rates was 1 beat (see Table 3).

**Table 3:** Comparison of our results with two previous studies in healthy individuals using commercial cycle ergometer

	This study	**Roest et al. (2001)**[5]	**Roest et al. (2004)**[6]
**n**	8	8	14

**Age (years)**	25.3 ± 4	17.5 ± 2.3	24.8 ± 5.2

**Weight (kg)**	67 ± 10	67 ± 12	74 ± 11

**Height (m)**	1.74 ± 0.07	1.74 ± 0.10	1.78 ± 0.06

**VO_2max _(mlO_2_/Kg/min)**	34.8 ± 5.4	39 ± 5	42 ± 5

**Rest heart rate (beats/min)**	67 ± 15	71 ± 10	67 ± 8

**Exercise**			

**Heart rate (beats/min)**	110 ± 1	121 ± 14	122 ± 8

**EDV (ml)**	142.4 ± 25.1	138 ± 27	148 ± 26

**ESV (ml)**	36.2 ± 10.2	36 ± 12	36 ± 14

**SV (ml)**	105.7 ± 18.7	102 ± 19	112 ± 15

**CO (L/min)**	11.6 ± 2.1	12.3 ± 2.3	

**EF (%)**	74.3 ± 4.7	74 ± 6	77 ± 6

**LVM (g)**	128 ± 28	-	133 ± 21

**Workload (W)**	96 ± 27	130 ± 21	132 ± 16

Data are mean ± SD. EF: ejection fraction; LVM: left ventricular mass; EDV: end-diastolic volume; ESV: end-systolic volume; SV: stroke volume; CO: cardiac output.

A disadvantage of the fixed resistance machine used in this study is that it is more difficult to establish a steady state workload. Indeed fixed workload machines are ideally engineered for this purpose (workload is unaffected by cadence). The fixed resistance machine used in this study is poorly suited for reproducing a relative workload (e.g.% maximal watts) because of the dependence on pedalling cadence. However, this can be readily achieved by using a metronome to provide a pedalling cadence at which any resistance can be used to develop a desired workload with minimal difficulty.

This MRI cycle ergometer demonstrated the value in obtaining MRI images during exercise at a fixed heart rate. In particular it enables identification of stroke volume which varied between 70-120 ml at rest increasing up to 200 ml during exercise, depending on ventricular morphology and fitness level [19-22]. As exercise intensity increases, stroke volume in physically unconditioned individuals increases gradually to a plateau at approximately 120 bpm [20], or 40% of $\dot{\text{V}}{\text{O}}_{2\text{max}}$[22]. Conversely, stroke volume continues to increase progressively until maximum heart rate in elite athletes [20,22,23]. The physiological responses obtained using our MRI-compatible cycle ergometer and protocols were comparable to previous studies in which an increase in heart rate and stroke volume increases cardiac output during exercise [7,8,14] (Table 3). Roest et al. used a commercial MRI cycle ergometer in healthy individuals (MRI cardiac ergometer, Lode BV, Groningen, The Netherlands) [7,8]. The first study examined left and right ventricular function in a group of healthy volunteers (mean age of 17.5 years) [7] (Table 3). The exercise images were obtained at 60% of maximal workload using 10 short-axis images for the left ventricular analysis. The second study involved individuals of similar age to our study participants, and the left ventricular volumes were obtained from 10 consecutive short axis scans, which were acquired during exercise at a workload corresponding to 60% of maximal oxygen consumption [8]. Even though the exercise workloads (approximately 130 W vs. 96 W) and heart rates (approximately 120 beats/min vs. 110 beats/min) were higher in their studies, the hemodynamic responses were similar. This is because Roest et al. participants were exercising at fixed workloads relative to their maximal capacity, while ours were exercising at a fixed heart rate of 110 beats/min. Moreover, our participants were comparatively less fit, requiring a lower workload to trigger an increase in heart rate.

Typically with MRI compatible cycle bikes the workload is either set at a constant level for all individuals or manually adjusted for each individual to increase and maintain heart rate at a predetermined criterion level such as 65% of maximum heart rate. According to Laperriere and colleagues 1989 a potential problem arises when manual adjustments of workload are used to produce and maintain specified heart rate. The workload value needed to maintain a predetermined heart rate percentage of maximum heart rate for a group of individuals, will be different for individuals with different pre-existing fitness levels [10]. Also, fatigue, even after a few minutes, could trigger a change in workload. Therefore, workload requires adjustments as heart rate is increased to the desired level, and may require continuous fine adjustments to maintain heart rate at target level [10].

In summary, the MRI-compatible cycle ergometer constructed by our research group allows for accurate and reproducible exercise cardiac assessments at tightly regulated heart rates, while continuously recording workload.

## Abbreviations

BMI: Body mass index; ECG: Electrocardiogram; EDV: End-diastolic volume; EF: Ejection fraction; EMI/RFI: Electromagnetic interference and radio-frequency interference; ESV: End-systolic volume; LVM: Left ventricular mass; MRI: Magnetic resonance imaging; RER: Respiratory exchange ratio; SV: Stroke volume; VO_2max_: Maximal oxygen consumption; VO_2_: Rate of oxygen consumption; VCO_2_: Carbon dioxide production.

## Competing interests

The authors declare that they have no competing interests.

## Authors' contributions

SG: conceived and designed the study, was involved in funding application, carried out data acquisition, analysis and interpretation, drafted and revised the manuscript. CS: was responsible for the software design. PH: participated in the coordination of the research group, involved in the acquisition of funding, participated in the writing and revision of the manuscript. WC: involved in the acquisition of funding, participated in the revision of the manuscript. JCB: involved in the acquisition of funding, participated in the revision of the manuscript. AT: participated in the design, and construction of ergometer and software interface; helped draft the manuscript. PN: participated in the design and construction of ergometer, supervised the bioengineering research group work, and helped draft the manuscript. All authors read and approved the final manuscript.
